# L-glutamine supplementation reduced morphological damage in the renal glomerulus of rats with Walker-256 tumor

**DOI:** 10.1590/acb383923

**Published:** 2023-10-13

**Authors:** Kaio Ramon de Aguiar Lima, Maria Luiza Diniz de Sousa Lopes, Sara Raquel Garcia de Souza, Luciane Fracaro, Natan Reyges Castro da Purificação, Marília Fabiana de Oliveira Lima, Lucas Alexandre Araújo Lins, Silvia Lacchini, Aurigena Antunes de Araújo, Raimundo Fernandes de Araújo, Juliana Vanessa Colombo Martins Perles, Jacqueline Nelisis Zanoni, Naianne Kelly Clebis

**Affiliations:** 1 Universidade Federal do Rio Grande do Norte – Natal (Rio Grande do Norte) – Brazil.; 2 Universidade Estadual de Maringá – Maringá (Paraná) – Brazil.; 3 Universidade de São Paulo – São Paulo (São Paulo) – Brazil.

**Keywords:** Kidney, Neoplasms, Antioxidants, Glutamine, Models, Theoretical

## Abstract

**Purpose:**

To evaluate the effects of the experimental subcutaneous Walker-256 tumor and L-glutamine supplementation, an antioxidant, on the glomerular morphology of rats.

**Methods:**

Twenty Wistar rats were distributed into four groups (n = 5): control (C); control treated with 2% L-glutamine (CG); rats with Walker-256 tumor (WT); and rats with Walker-256 tumor treated with 2% L-glutamine (WTG). Renal histological samples were submitted to periodic acid-Schiff and Masson’s Trichrome staining to analyze glomerular density, morphometry of glomerular components and glomerulosclerosis; and to immunohistochemistry for fibroblast growth factor-2 (FGF-2).

**Results:**

WT showed 50% reduction in body mass gain and cachexia index > 10%, while WTG demonstrated reduction in cachexia (*p* < 0.05). WT revealed reduction of glomerular density, increase in the glomerular tuft area, mesangial area, matrix in the glomerular tuft, decrease in the urinary space and synechia, and consequently higher glomerulosclerosis (*p* < 0.05). L-glutamine supplementation in the WTG improved glomerular density, and reduced glomerular tuft area, urinary space, mesangial area, and glomerulosclerosis compared to WT(*p* < 0.05). WT showed higher collagen area and FGF-2 expression compared to C (*p* < 0.05). WTG presented lower collagen fibers and FGF-2 expression compared to WT (*p* < 0.05).

**Conclusions:**

L-glutamine supplementation reduced cachexia and was beneficial for glomerular morphology of the rats, as well as it reduced kidney damage and improved the remaining glomeruli morphology.

## Introduction

Cancer is no longer considered just a mass of cells produced by uncontrolled proliferation, but it is rather a complex tissue composed of multiple distinct cell types that communicate by heterotypic interactions with one another^1^, and it is closely associated to oxidative stress and inflammation^2^. Most patients with cancer develop cachexia^3^,^4^, a multifactorial metabolic imbalance, influenced by the activation of pro-inflammatory cytokines which lead to weight loss, mainly by reducing muscle mass and adipose tissue^5^-^7^.

In cachexia, mitochondrial damage occurs, which increases the production of reactive oxygen species (ROS), resulting in oxidative stress associated with a deficit of antioxidant activity^8^,^9^. The kidneys are highly vulnerable to damage caused by ROS^9^. After a kidney injury, during the tissue repair stage, fibrosis may occur, promoting changes in the mesangial cells and matrix, and glomerular basement membrane^10^,^11^. In patients with solid tumors, a few renal changes are observed; however, cancer patients with cachexia may present more significant renal changes, since the loss of muscle mass reduces the serum creatinine levels, which can modify the dosages of drugs excreted by the kidneys^12^.

Substances that activate antioxidant pathways have been investigated, such as L-glutamine, a precursor of glutathione, the main non-enzymatic cellular antioxidant of the body^13^. L-glutamine is also a source of energy for proliferating enterocytes, assisting in the maintenance of the intestinal barrier^14^,^15^, and providing energy for immune cells^16^.

L-glutamine supplementation has been evaluated in pathological processes with catabolic stress, including cancer, as it can act as an important modulator^17^,^18^. In cancer, the reduction of glutamine can stimulate the release of extracellular macrovesicles with pro-tumorigenic functions related to tumor adaptation to stress^17^. However, there is no consensus in the literature regarding the role of glutamine in cancer cells, as some studies indicate that glutamine serves as a substrate for malignant cells^19^,^20^ in tumors located in the pancreas, intestine, and spleen, while others indicate that glucose and lactate are substrates for tumor cells in the brain, lung, and other tissues^21^,^22^.

In the present study, the experimental model of Walker-256 carcinosarcoma was used to mimic cachexia and immunosuppression conditions^23^, promoting extensive weight loss due to carbohydrates, lipids, and proteins catabolism^24^, despite the great aggressiveness and rapid growth of the tumor, which ultimately cause the death of the rats in a maximum of 15 days^25^.

In this sense, this study aimed to evaluate the effect of L-glutamine supplementation on the glomerular morphology of rats with Walker-256 tumor.

## Methods

### Animals

Kidneys were removed from the carcasses of 20 Wistar rats (*Rattus norvegicus*), males, aged 64 days old, obtained from the Central Animal Facility of the Universidade Estadual de Maringá. All procedures are in accordance with ethical principles adopted by the Brazilian College of Animal Experimentation and approved by the Ethics Committee on Animal Use of Universidade Federal do Rio Grande do Norte (Protocol No. 066/2016).

During the 14-day experimental period, the rats were kept in polypropylene boxes in the animal experimentation room of the Department of Morphological Sciences of the Universidade Estadual de Maringá, under controlled environmental conditions of temperature (22° ± 2°C) and luminosity (12-h light/12-h dark cycle), with food and water *ad libitum*.

The rats were randomly (simple random sampling) assigned to four groups (n = 8 rats/group), as follows:

- Control without supplementation (C);
- Walker-256 tumor-bearing rats without supplementation (WT);
- Control supplemented with 2% L-glutamine (CG);
- Walker-256 tumor-bearing rats supplemented with 2% L-glutamine (WTG).

The rats were weighed after tumor induction and at the end of the experiment. Three deaths occurred in each WT and WTG groups. Therefore, we kept five rats per group (controls and experimental groups).

### Walker-256 tumor cells

Walker-256 tumor cells were maintained with weekly passages by aseptic intraperitoneal inoculation of 2 × 10^6^ cells per rat. After seven days of ascitic growth, the rats were euthanized. Peritoneal exudate was removed and submitted to centrifugation to obtain the tumor cells. The tumor cells were resuspended in phosphate buffered saline (PBS) (16.5 mM, pH 7.5) for counting in the Neubauer chamber. Tumor cells viability was evaluated by the trypan blue exclusion assay^26^.

In the WT and WTG groups, rats were injected in the right rear flank, a 0.5-mL tumor cell suspension containing 8 × 10^7^ viable cells in PBS (16.5 mM, pH 7.5). The C and CG rats were inoculated with PBS (16.5 mM, pH 7.5) in the identical area to promote the same stress suffered by the rats with tumor^26^.

### Treatment with 2% L-Glutamine

The supplemented rats received L-glutamine (Deg Importação de Produtos Químicos LTDA, São Paulo, SP, Brazil) incorporated into the standard feed in the proportion of 2 g/100 g of feed (Nuvilab, Colombo, PR, Brazil). The feed was ground, added to L-glutamine (Fagron do Brasil Farmacêutica LTDA, São Paulo, SP, Brazil), then reconstructed into pellets, which were later dried in the laboratory oven^27^. Non-supplemented rats received balanced standard rodent chow.

### Material collection and processing

At the end of the experiment, all rats were weighed and euthanized with thiopental (40 mg/kg of body weight; Abbott Laboratories, Chicago, IL, United States of America), intraperitoneally administered. The Walker-256 solid tumor was removed and weighed. A laparotomy was performed to collect the kidneys, which were washed in PBS, weighed, submitted to fixation, and destined for processing.

After 48 hours of fixation in 10% buffered formalin solution, the kidneys were coronally sectioned, and the specimens were destined for histological routine with dehydration in an increasing series of alcohols (80%, 90%, absolute I, II and III), clearing in xylene (three times) and embedded with paraffin. Slides with 3-μm thick sections were obtained and destined periodic acid-Schiff (PAS), Masson’s trichrome, and immunohistochemical staining^28^.

### Cachexia index

To assess the cachexia index (CI), the total tumor mass (flank tumor and metastases) was dissected and weighed. The results were used in Eq. 1, and rats with a loss of body mass greater than 10% were considered cachectic^26^:


$$\text{Cachexia index}\%=\frac{\left(IBW-FBW+TW+BWG\right)}{IBW+BWG}\times 100$$ (1)

Where: IBW: the initial body weight of the tumor-bearing rat; FBW: the final body weight of the tumor-bearing rat; TW: the tumor weight; BWG: the mean body weight gain of the C group.

### Histological analysis

For PAS staining, the slides were dewaxed (xylene) and hydrated through absolute alcohol up to 80% alcohol and with distilled water (3 min). Samples were stained with 1% periodic acid (15 min, in refrigerator), washed in distilled water (3 min), and immersed in the Schiff’s reactive solution (1 hour, in the dark and in the refrigerator). The slides underwent three baths in sulfuric water (3 min) and were washed with running water for 10 min, stained with hematoxylin for 40 sec and were washed again in running water (10 min). Slides were then dehydrated in an increasing series of alcohols, clarified in xylene, and mounted with a cover slip and Permount mounting medium (Synth, Diadema, SP, Brazil).

For Masson’s trichrome staining, after deparaffinization and rehydration, as described before, the slides were washed with distilled water for 1 min and placed in Boiün’s solution overnight in an oven (60°C). The samples were washed in running water until the yellow of the fixative disappeared, and in distilled water to initiate the staining. The slides were stained for 10 min in Weigert’s ferric hematoxylin solution (A and B), washed in running water (10 min) and in distilled water. Next, they were stained with Biebrich’s Scarlet solution (5 min) and quickly washed in distilled water. The slides were then placed in the phosphotungstic/phosphomolybdic acid differentiation solution for 10–15 min and washed in distilled water. The samples were stained by the aniline blue solution (5–10 min) and washed in distilled water. They were placed in 1% glacial acetic acid solution (3–5 min), washed in distilled water, dehydrated, clarified, and evaluated.

Renal histological analysis was blindly performed by one evaluator. The slides were coded to avoid the identification of the rat/group during the analysis. After measurements, the data were tabulated, and the groups revealed for the statistical analysis.

### Glomerular density and morphometric analysis of the glomerular components

PAS-stained slides were evaluated regarding the numerical density of the renal glomeruli (glomeruli/mm^2^) present in 20 microscopic fields per rat. Microscopic fields were determined using the 10x objective of the Nikon Elipse E200-LED microscope with a Nikon digital camera DXM 1200, at the Laboratory for Research on Cancer and Inflammation, Department of Morphology, at the Universidade Federal do Rio Grande do Norte (microscopic field area = 0.8265 mm^2^; total area analyzed per rat = 16.53 mm^2^).

**Figure 1:**
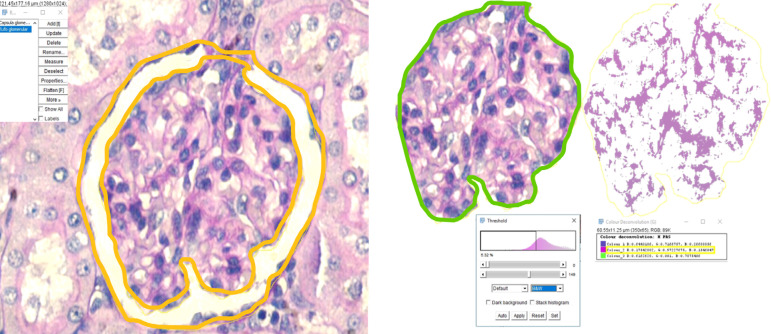
Image processing method for morphometric analysis of renal components. Determination of glomerular tuft area, glomerular corpuscle area, mesangial matrix area and mesangial matrix index in the glomerulus with ImageJ software (PAS, 40x).

Source: Elaborated by the authors. PAS: periodic acid-Schiff.

These slides were also used for semi-quantitative measurement of the glomerulosclerosis index^29^, glomerular area (μm^2^), glomerular tuft area (μm^2^), area of the urinary space (μm^2^), mesangial area (μm^2^), and the glomerular tuft area (%) occupied by extracellular matrix in 20 glomeruli per rat. Glomerular components were evaluated under a 40x objective (microscopic field area = 0.05165 mm^2^; total area analyzed per rat = 1.0331 mm^2^), using the ImageJ software. Freehand selections tool in ImageJ was applied to demarcate and measure the glomerular capsule area, followed by the tuft area, allowing to extract the urinary space area as a result. In the same capture, within the selection of the glomerular tuft, a color deconvolution was performed using the vector H-PAS (*Image > Color > Colour Deconvolution >* HPAS) to measure the mesangial area. The mesangial matrix index was determined by the proportion of the glomerular tuft occupied by the mesangial matrix, excluding the nuclei (Image > Adjust > Threshold > Select mesangial markup limit > Apply > tuff selection > Measure) (Fig. 1)^28^,^30^.

PAS-stained slides were also used to assess the glomerulosclerosis using a semi-quantitative technique. The degree of sclerosis in each glomerulus was subjectively classified as follows:

- Grade 0: normal;
- Grade 1: slightly sclerotic, sclerotic area of the glomerular tuft ≤ 25%;
- Grade 2: moderately sclerotic, sclerotic area: 25–50%;
- Grade 3: severely sclerotic, sclerotic area: 50–75%;
- Grade 4: globally sclerotic, sclerotic area: 75–100%.

Glomerulosclerosis was defined as thickening of the glomerular basement membrane, mesangial hypertrophy, capillary occlusion, and synechia of the glomerulus adherent to the glomerular capsule. The glomerulosclerosis index was determined by the percentage of glomeruli score per group^28^,^30^.

### Renal collagen

**Figure 2:**
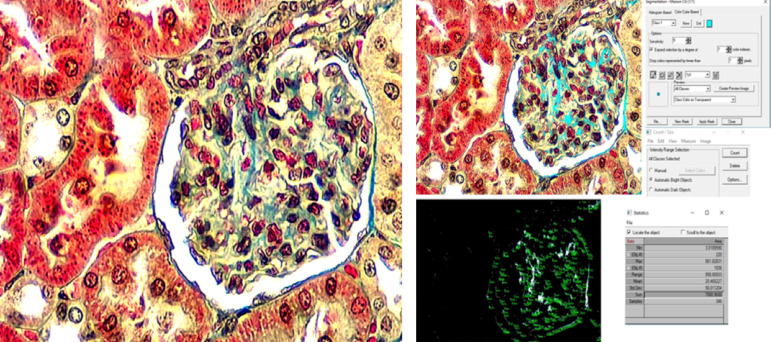
Image processing method for morphometric analysis of renal components. Determination of the area of collagen fibers in the glomeruli with ImagePro Plus 6.0 software (Masson’s trichrome, 40x)

Source: Elaborated by the authors.

Evaluation of the area occupied by collagen was performed on the samples stained with Masson’s trichrome with a 40x objective magnification, using the Image Pro Plus 6.0 software (Ipwin 32, Media Cybernetics, L.P., MD, United States of America). In each field, the area stained in blue was selected and measured (*Measure > Count/Size > SelectColors... > Precision1X1* > All collagen area was marked > *NewMask > Close > AutomaticBrightObjects > Count > View > Statistics* > copy the amount -*Sum*-) (Fig. 2).

### Immunohistochemistry

Immunohistochemical reactions were performed to detect fibroblast growth factor-2 (FGF-2) in the kidney tissue. The slides were deparaffinized in two xylene washes (10 min) and passed in three baths (3 min) in absolute, 95 and 80% alcohol washes to remove the excess of xylene, followed by hydration in distilled water. Slides were immersed in PBS for 10 min, and antigen retrieval was performed with sodium citrate solution in a water bath (30 min). Sections were immersed in 3% hydrogen peroxide solution for blocking endogenous peroxidase activity (Synth, Diadema, SP, Brazil) for 20 min. Next, sections were incubated with 5% bovine serum albumin (BSA, Sigma, St. Louis, MO, United States of America) diluted in PBS for 2 hours to block nonspecific staining. Two washes were performed with distilled water and two with PBS, followed by incubation with primary anti-FGF-2 antibody (1:800; sc-1360, Santa Cruz Biotechnology, Interprise, Brazil), overnight. The material was washed with PBS and incubated with secondary antibody conjugated to streptABComplex/HRP Duet, Mouse/Rabbit (K0492 DAKO, Dako North America, Carpinteria, CA, United States of America), at room temperature. Two washes with PBS were performed, and the DAKO Liquid DAB + substrate Chromogen System kit (K3468 DAKO, Dako North America, Carpinteria, CA, United States of America) was applied (10 min). Counterstained with Harris Hematoxylin (MHS16-Sigma, Sigma, St. Louis, MO, United States of America) was performed. The material was dehydrated in 95% alcohol and absolute alcohol (three washes) for 10 sec each, and in xylene (three washes, 10 sec), and mounted with Permount mounting medium.

Stereological analysis of volume density (*V*_V_) was carried out to evaluate the expression of FGF-2^29^. In the previously captured images, a test system consisting of 475 points was applied and Eq. 2 was used:


$$VV=\frac{P_{\text{int}}}{P_{\text{ref}}}$$ (2)

Where: P_int_: the total number of points that touched the cells of interest; P_ref_: the total number of test system points (475).

### Statistical analysis

The results were expressed as mean ± standard error of the mean (SEM). Analysis of variance (ANOVA) was used, followed by Tukey’s test (GraphPad Prism 6 Software, La Jolla, CA, United States of America). Data regarding tumor mass and CI were analyzed by Student’s *t*-test. The glomerulosclerosis index was statistically analyzed using the χ^2^ test. A significance level of 5% was adopted for all tests.

## Results

### Physiological parameters

Table 1 shows the physiological parameters of the sample. Comparing the final body weight of the rats in the controls to the tumor groups, no statistically significant difference was observed (*p* > 0.05). In WTG, there was a 7.87%-lower (38.53 ± 3.34 g) tumor weight compared to WT group (41.82 ± 1.08 g), but this difference did not reach statistical significance (*p* > 0.05). As for body weight gain, a significant 50.56%-reduction in the WT group and 29.59%-reduction in WTG were observed when compared to C group (*p* < 0.05); however, no differences were observed between the control groups (C × CG) and between the tumor groups (WT × WTG) (*p* > 0.05). The CI for the WT group was 10.83 ± 2.29%, while WTG group exhibited a CI of 5.20 ± 2.58% (*p* < 0.05). The weight of the right and left kidneys was not statistically different between groups (*p* > 0.05).

### Glomerular density

**Figure 3:**
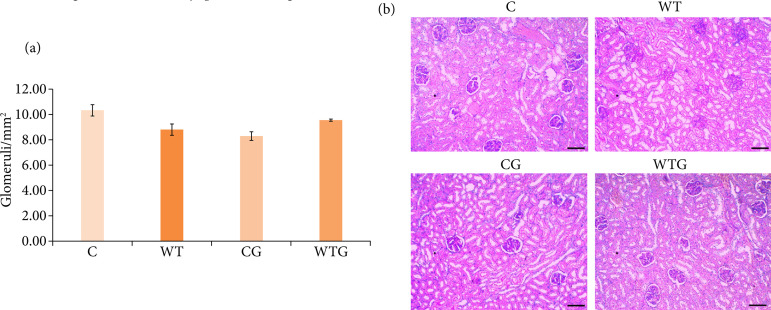
Glomerular density analysis. **(a)** Glomerular density (glomeruli/mm^2^) of C, WT, CG and WTG groups (n=5/group). Different letters indicate statistically significant difference (*p* < 0.05) by Tukey’s test (n = 5). **(b)** Representative photomicrographs of the C, WT, CG and WTG groups (PAS, 10x, scale bar: 50 μm).

Source: Elaborated by the authors. C: control; CG: control treated with 2% L-glutamine; WT: rats with Walker-256 tumor; WTG: rats with Walker-256 tumor treated with 2% L-glutamine; PAS: periodic acid-Schiff.

Glomerular density in the WT (16.95%) and CG (19.59%) was lower compared to C group (*p* < 0.05). In the rats of the WTG group, there was 7.87% higher glomerular density compared to WT. Compared to C group, the WTG rats showed 7.21% lower glomerular density (*p* > 0.05) (Fig. 3).

### Morphometry and glomerulosclerosis

**Figure 4:**
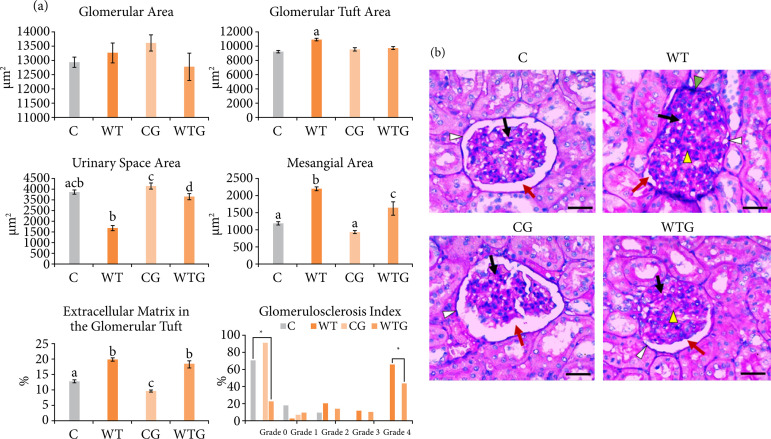
Glomerular components. **(a)** Graphs illustrating the morphometric analysis of the glomerular components in the C, WT, CG and WTG groups (n = 5/group). Different letters indicate statistically significant difference (p < 0.05) by Tukey’s test, and * indicates statistically significant difference (p < 0.0001) by χ^2^ test. **(b)** Representative photomicrographs of the C group: glomeruli with normal morphology, glomerular capsule (white arrowhead), urinary space (red arrow) and glomerular tuft (black arrow); WT group: glomeruli with morphometric changes, synechia of the glomeruli adherent to the glomerular capsule (green arrowhead), basement membrane thickening and mesangial hypertrophy (yellow arrowhead); CG and WTG groups showing glomerular capsule (white arrowheads), urinary space (red arrow), glomerular tuft (black arrow). Glomerulus with mesangial hypertrophy (yellow arrowhead) in the WTG group (PAS, 40x, scale bar: 30 μm).

Source: Elaborated by the authors. C: control; CG: control treated with 2% L-glutamine; WT: rats with Walker-256 tumor; WTG: rats with Walker-256 tumor treated with 2% L-glutamine; PAS: periodic acid-Schiff.

Morphometric analysis of the glomerular components and glomerulosclerosis are shown in Fig. 4. No significant differences were found between groups regarding the glomerular area (*p* > 0.05). In the WT group, results showed a 19.09%-higher glomerular tuft area compared to C, and a 11.49%-higher glomerular tuft area than WTG group (*p* < 0.05). Comparison between the glomerular tuft area of C, CG and WTG groups showed no significant differences. A 50.73%-lower urinary space area value was found in WT rats compared to the C group (*p* < 0.001). In WTG, there was a 47.13%-larger urinary space area compared to WT rats (*p* < 0.001), as well as a similar area compared C group.

Synechia was observed in 80% of the evaluated glomeruli of the WT rats, in which the tuft was attached to the glomerular capsule, with total or partial absence of the urinary space (Fig. 4, black arrowhead). This feature was not observed in WTG, suggesting a beneficial effect of L-glutamine supplementation on glomerular integrity.

WT rats showed a 46.39%-higher mesangial area compared to C group (*p* < 0.001). In WTG, there were a 26.25%-lower mesangial area compared to WT (*p* < 0.01) and a 27.30%-higher mesangial area compared to C group (*p* < 0.05). The CG group showed a 21.38%-lower mesangial area than C group, but this difference was not statistically significant (*p* > 0.05).

The percentage of the glomerular tufts occupied by extracellular matrix in WT rats was higher compared to C group (*p* < 0.001). WTG showed a 30.5%-increase in this parameter compared to C group (*p* < 0.001). In CG group, a 24.84%-lower percentage of the glomerular tufts occupied by extracellular matrix was verified when compared to C rats (*p* < 0.05).

Evaluation of the glomerulosclerosis index (Fig. 4) revealed that 72% of the glomeruli in the C group was classified as grade 0, 28% were classified among grade 1-2, and none of them were graded as grade 3-4. In the CG, there were a 22.5%-increase in the grade 0 glomeruli and a 75%-reduction of those classified as grade 1 in relation to C group (*p* < 0.05). In CG group, no glomerulus was classified as grade 2–4, suggesting a beneficial effect of L-glutamine supplementation on the maintenance of the glomeruli in these rats. In WT, 67% of the glomeruli were grade 4, 11% were grade 3 and 22% were grade 1-2, whereas no glomerulus was classified as grade 0. In WT group, 77% of the glomeruli showed glomerulosclerosis (grades 3-4) *versus* 0% in the C group (*p* < 0.05). WT also presented a 78%-reduction in healthy glomeruli compared to C group (*p* < 0.0001). In the WTG group, a 34.28%-reduction in the glomeruli identified as grade 4 was verified (*p* < 0.001), as well as a 56.52%-increase in the grade 0–2, compared to WT group (*p* < 0.0001), indicating a protective effect of L-glutamine in the glomeruli of the rats with cancer.

### Collagen fibers and immunoexpression of FGF-2

**Figure 5:**
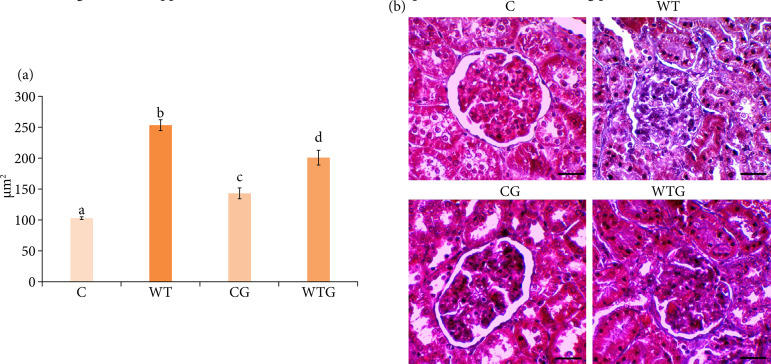
Analysis of glomerular fibrosis. **(a)** Area occupied by collagen fibers in the renal glomeruli (μm^2^) of the C, WT, CG and WTG groups (n = 5/group). Different letters indicate a statistically significant difference by Tukey’s test (*p* < 0.05). **(b)** Representative photomicrographs of glomerular collagen fibers of the C, WT, CG and WTG groups (Masson’s trichrome, 40x, scale bar: 20 μm).

Source: Elaborated by the authors. C: control; CG: control treated with 2% L-glutamine; WT: rats with Walker-256 tumor; WTG: rats with Walker-256 tumor treated with 2% L-glutamine.

**Figure 6:**
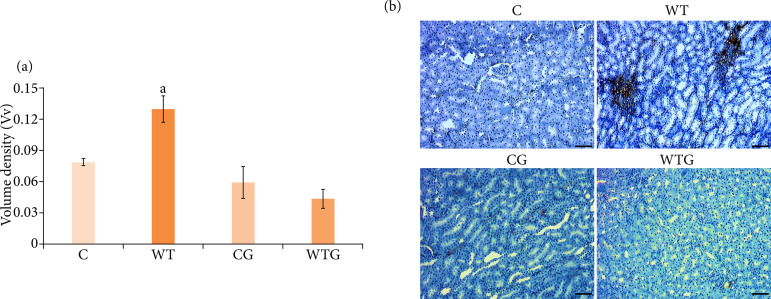
Immunoexpression of FGF-2 in the renal tissue from cachectic rats. **(a)** Volume density (*V*_V_) of samples submitted for immunohistochemical analysis of FGF-2 in the C, WT, CG and WTG groups (n = 5/group). Different letters indicate statistically significant difference by Tukey’s test (*p* < 0.05). **(b)** Representative photomicrographs of expression of FGF-2 in renal rats of the C, WT, CG and WTG groups (FGF-2, 10x, scale bar: 50 μm).

Source: Elaborated by the authors. C: control; CG: control treated with 2% L-glutamine; WT: rats with Walker-256 tumor; WTG: rats with Walker-256 tumor treated with 2% L-glutamine; FGF-2: fibroblast growth factor-2.

Renal fibrosis was evaluated by the area occupied by collagen fibers in the renal glomeruli (Fig. 5) and by the immunoexpression of FGF-2 (Fig. 6). A 59.32%-increase of glomerular collagen was observed in the WT group compared to C (*p* < 0.001). In WTG, we found 20.29%-reduction of collagen fibers when compared to WT group (*p*<0.001); however, it was 48.83%-higher in WTG compared to C group (*p* < 0.001). In CG group, collagen fibers showed 28.29%-higher values than those found in C group (*p* < 0.01).

In WT, a 39.47%-higher immunoexpression of FGF-2 was found when compared to C group (*p* < 0.05). In WTG, there were a 66.91%-decrease in FGF-2 expression compared to WT (*p* < 0.001) and a 45.33%-decrease when compared to C group (*p* < 0.05).

In WT, there was an increase in collagen deposition and FGF-2 expression, which together with the glomerulosclerosis index, indicated a process of tissue fibrosis, while in WTG the findings indicate reduction in these indexes, suggesting benefits of L-glutamine supplementation in rats with cancer and point to a tissue remodeling process.

## Discussion

In this study, supplementation with L-glutamine reduced the cachexia and was beneficial for glomerular morphology of the rats. We also observed a decrease in the tumor mass of rats treated with L-glutamine, but this reduction was not significant. In a study with liver cancer, the authors also found no changes in tumor weight^4^; however, in a previous study conducted by our research group, a significant reduction in tumor mass was observed due to L-glutamine supplementation in the rats with tumor^31^.

Weight loss is higher in cancer patients, as tumors cause excessive energy consumption and generate energetic inefficiency, being accountable for an increase in the glucose usage, and a depletion in intramuscular and hepatic glycogen^4^,^31^. The loss of body mass in cachexia is related to reduced muscle and adipose mass due to inadequate neurohormonal anabolic activity and/or excess catabolic activity^5^-^7^.

In this study, no changes were identified in the final weights of the rats. However, when analyzing the body weight variation, we observed a reduced weight gain in the WT group, which occurred along with a higher rate of cachexia than in the WTG rats, indicating a beneficial effect of L-glutamine supplementation. A delay in body weight loss has been observed with the use of rosiglitazone^6^. Salidroside administration reduced cachexia, leading to less weight loss and less tumor mass^32^. In the same way, pioglitazone treatment in rats inoculated with Walker-256 tumor cells increased the survival time of cachectic rats due to greater maintenance of body mass and reduced tumor mass^33^.

A previous study evaluated the blood cortisol levels, glucose, insulin, and urea in rats with Walker-256 tumor with and without 2% L-glutamine supplementation for 10 days and found no changes in cortisol and urea levels between control rats (with and without supplementation) and rats with tumor (with and without supplementation)^31^. However, the authors reported an increase in the glucose and insulin levels of the tumor-bearing rats treated with 2% L-glutamine, suggesting a positive effect of supplementation on energy balance, with reduced tumor mass and decreased cachexia, probably due to the insulin stimulation activity on lipid and protein anabolism^31^.

The ROS production is a physiological process. However, imbalance between the generation of ROS and endogenous antioxidants levels results in oxidative stress^8^,^9^. The effects of antioxidant therapy in treating cancer, especially with glutamine, are controversial^17^,^18^. However, previous studies suggest that 2% L-glutamine supplementation in the Walker-256 experimental tumor model may help to reduce ROS levels and inflammation, and may consequently be beneficial to the body^21^,^22^,^31^.

Cachexia may worsen kidney function in patients with chronic kidney disease^12^, since oxidative stress and vascular resistance induce changes on the renal perfusion levels. In this study, we observed that supplementation with 2% L-glutamine reduced the loss of glomeruli in the WTG compared to the WT group, and changes in the glomeruli number could compromise body homeostasis, impairing renal filtration. In a study analyzing the urinary proteomics using urine samples collected on different days after inoculation of Walker-256 tumor cells in rats, alterations in the pattern of 10 urinary proteins were found with the development of cancer, which occurred before the tumor mass was palpable^34^. This could allow early detection of cancer or serve as an index for evaluating tumor progression, however the authors did not evaluate the renal morphology of the rats.

In addition to changes in glomerular density, we also observed glomerular tuft hypertrophy, perhaps produced by mesangial proliferation and mesangial matrix proliferation in the glomerulus, synechia and reduction of urinary space in the WT group. Also, 77% of the glomeruli in this group were severely compromised, as they showed a sclerotic area greater than 50%, indicating glomerulosclerosis, while the WGT group presented lower values, validating that 2% L-glutamine supplementation has a beneficial impact on the evaluated parameters.

Glomerular and tubular cells react to the inflammatory process with basement membrane lesion and with epithelial-mesenchymal transition, thereby converting the cells into fibroblasts with the ability to produce collagen, which in turn can cause damage to the renal vessels, tubules, and glomeruli, leading to renal fibrosis^35^, stimulated by the action of FGF-2^36^.

In diseases which promote oxidative stress and inflammation, diminishing the renal function, the histological findings usually indicate necrosis and tubular fibrosis, but structural changes in the glomeruli may occur, as they are the morphofunctional units of the kidneys. Endothelial cells, podocytes and mesangial cells stand out in the glomeruli constitution, perform blood filtration, and produce primary urine through the glomerular filtration barrier activity^37^,^38^.

A study evaluating kidney changes related to diabetes showed glomerular hypertrophy, and increased accumulation of type IV collagen and mesangial matrix^39^. Mesangial matrix is formed by different cells among types IV and V collagen fibers which can increase in pathological processes, and consequently lead to mesangial expansion and glomerular sclerosis^37^. This could explain our findings, since the mesangial changes, the accumulation of collagen fibers and high FGF-2 expression were accompanied by marked glomerulosclerosis and a decrease in glomerular density in the WT group, which was partially reversed by 2% L-glutamine supplementation.

This higher incidence of glomerulosclerosis in the WT group can be attributed to the inflammation and local oxidative stress process^2^,^9^. Nevertheless, 2% L-glutamine was able to minimize this devastating effect on the kidneys of WTG rats, possibly due to its immunomodulatory activity, acting as fuel for immune cells, and as a precursor for glutathione synthesis, which is an important antioxidant enzyme^16^. Therefore, the recovery in adequate plasma glutamine levels probably lowered the physiological changes that trigger and worsen the cachexia process^14^.

We identified a significant decrease in collagen deposition in the WTG group compared to WT, perhaps due to the reduced inflammation with the invigoration of the immune system associated with the ROS removal mediated by glutathione^13^-^16^. In addition, a reduction in the FGF-2 present in the tissue was observed in the WTG rats, constituting an important result of treatment with 2% L-glutamine as it may indicate a pathological process in the glomerulus suggestive of kidney damage, which is a common finding in chronic kidney diseases^40^.

## Conclusion

The experimental cancer model by inoculating Walker-256 tumor cells caused cachexia in the rats. Significant glomerular changes such as synechia, increased glomerular tuft area, reduced urinary space, increased mesangial area and mesangial matrix, increased collagen deposition and FGF-2 expression were observed, which were accompanied by a high glomerulosclerosis rate and reduced glomerular density in tumor-bearing rats without treatment. In contrast, supplementation of tumor-bearing rats with 2% L-glutamine had a beneficial effect by reducing the cachexia, and it reversed or minimized the glomerular lesions at the renal level seen in the rats with tumor. However, other studies using this experimental model are needed to elucidate the role of glutamine in the other components of the renal parenchyma.

## Data Availability

The data will be available upon request.
